# Posture Monitoring During Breastfeeding: Smart Underwear Integrated with an Accelerometer and Flexible Sensors

**DOI:** 10.3390/s24237641

**Published:** 2024-11-29

**Authors:** Beibei Zhou, Ruixin Liang, Jun Zhang, Xiaolu Li, Zowie Broach, Joanne Yip

**Affiliations:** 1 School of Fashion and Textiles, The Hong Kong Polytechnic University, Hung Hom, Kowloon, Hong Kong SAR, China; beibei.zhou@polyu.edu.hk (B.Z.); ruixin.liang@polyu.edu.hk (R.L.); alice.zj.zhang@connect.polyu.hk (J.Z.); xiao-lu.li@connect.polyu.hk (X.L.); 2 Design Department, Royal College of Art, London SW7 2EU, UK; zowie.broach@rca.ac.uk

**Keywords:** breastfeeding posture monitoring, smart nursing underwear, ergonomic sensor integration, musculoskeletal pain

## Abstract

The position that a woman adopts during breastfeeding is important for both infant and maternal health; however, many women experience musculoskeletal pain due to poor posture during breastfeeding, which is a known factor in low exclusive breastfeeding rates. Posture monitoring is an effective intervention, but existing wearable devices do not consider the ergonomics of nursing mothers and breastfeeding scenarios. In this study, nursing underwear was developed with posture monitoring and a real-time feedback system using accelerometers and flexible bending sensors targeting the neck and upper thoracic spine. Semi-structured interviews were conducted with 12 Chinese mothers to identify key challenges and inform the design. After designing and producing the prototype, wear trials were conducted with two participants who tested both the prototype and a commercial sample while holding a 4 kg baby doll. Video recordings and questionnaires were used to assess the underwear’s effectiveness. The results showed improvements in postural alignment and an increase in the frequency and duration of relaxation periods. Participants reported that the prototype surpassed the commercial sample in functionality, comfort, and aesthetics. These findings are significant for postpartum health and provide guidelines for future smart nursing garment development.

## 1. Introduction

According to the United Nations Children’s Fund (UNICEF), over 130 million newborns are born globally each year [1]. The World Health Organization recommends that babies should be exclusively breastfed for at least six months and then, along with complementary food for up to two years or beyond [2]. However, breastfeeding often requires long periods of maintaining a static posture, as the feeding sessions can last up to 30 min and occur up to 10 times daily [3]. Consequently, poor posture during breastfeeding can lead to a range of musculoskeletal disorders, including mechanical neck pain, lower back pain, scapular dyskinesia, carpal tunnel syndrome, and even sciatica [4,5,6]. Moreover, these physical issues can also impact maternal mental health, which may indirectly affect infant development [7,8]. Although improper posture during breastfeeding can cause significant pain, many nursing mothers have little knowledge and awareness of the correct posture for breastfeeding. Farooq et al. [4] conducted a cross-sectional study in Pakistan and reported that 43.3% of the women in their study had poor knowledge about the correct positions for breastfeeding. People with less sensitive internal systems would maintain an incorrect posture for extended periods of time until their symptoms become severe enough to require medical attention [9]. Therefore, smart systems that address improper posture during breastfeeding and provide external feedback are essential.

A number of solutions have been proposed in the literature to mitigate this issue. For example, Afshariani et al. [10] found that interventions and training in the use of different positions during breastfeeding can relieve musculoskeletal pain. However, this solution requires long-term intervention and follow-up, which is time-consuming and labor-intensive. Current products that assist with positioning the mother during breastfeeding, such as breastfeeding pillows and chairs, are not easily portable and still require the users themselves to have an awareness of ergonomic principles [11,12]. Convenient and portable postural assistive products, such as underwear with orthopedic features, can effectively alleviate postural problems [13,14], but their structure does not facilitate breastfeeding.

In recent years, wearable technology has matured and is being used in various healthcare domains, particularly for posture monitoring [15]. More recently, inertial measurement unit (IMU) sensors, which are known for their good accuracy in motion tracking, low power consumption, real-time data capabilities, and lightweight design, have gained significant attention and have been increasingly integrated into clothing for posture detection. Bootsman et al. [16] proposed the BackUp system, which is designed to monitor lumbar posture and incorporates two IMU sensors that are placed in pockets located on the lower back of the smart shirt. This system has shown positive results in lumbar posture monitoring since the focus is on the lumbar curvature rather than the tilt angle, thereby mitigating the risk of false readings. Its design closely resembles ordinary clothing, thus minimizing additional discomfort and significantly altering daily wear habits. However, the system in Bootsman et al. [16] does not comprehensively monitor the upper body nor cover key areas relevant to posture during breastfeeding, such as the thoracic spine, neck, and shoulders. Tlili et al. [17] developed a posture-tracking system in the form of a smart belt, which has the advantage of comprehensive trunk monitoring, including the shoulders, thus providing complete information on the back posture. Nonetheless, the smart belt is relatively bulky and thick, so it lacks the necessary aesthetic appeal for external wear or the wear comfort required for close-to-body use. Furthermore, this design does not account for the potential issues of layering over the bra, which could diminish the overall user experience. Abro et al. [18] developed a knitted flexible smart garment (FSG) for posture detection, which uses thin, safe, and low-cost sensors that change resistance when bent, thus making them ideal for monitoring human joints. The study demonstrates the potential of using flexible sensors in developing smart, close-fitting garments as they are lightweight and soft, which support a good wear experience. The monitoring system successfully detects different joint flexion positions; however, the study is limited as there is only one male participant, which significantly restricts the generalizability of the results. Additionally, the FSG is not washable, which limits its practical application. Overall, these studies have achieved positive results in posture monitoring technology yet have not considered the ergonomics of nursing mothers and postpartum usage scenarios in their design, materials, and interaction methods. In addition, women who are breastfeeding and need to wear posture-assisting products also have to accommodate their underwear, which increases the inconvenience.

Therefore, this study is conducted in response to these gaps by developing multifunctional nursing underwear that incorporates posture monitoring, postural support, and breastfeeding accessibility features. This intelligent underwear provides real-time feedback on breastfeeding posture, which helps to reduce the chances of incurring musculoskeletal pain and facilitates postnatal postural recovery. The design is anticipated to increase the breastfeeding rate and improve infant health outcomes.

## 2. Materials and Methods

**Figure 1:**
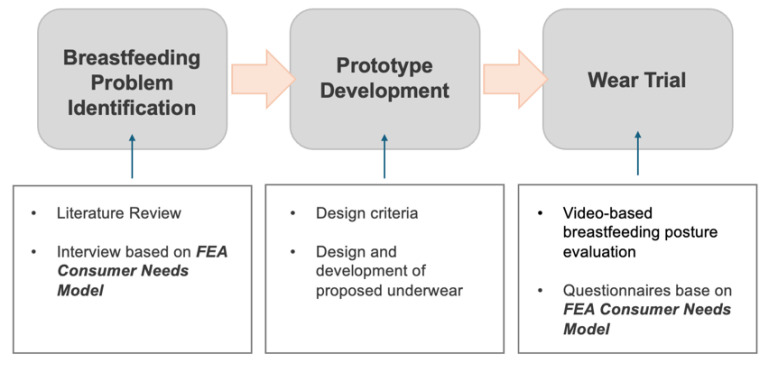
*Overview of study design.* Created by the authors.

This study adopts a mixed methods approach throughout the design process, which comprises three key phases: identification of breastfeeding and postpartum issues and needs; designing and development of the smart nursing underwear; and evaluation of the effectiveness of the proposed underwear (Figure 1). First, in-depth interviews with 12 Chinese mothers who were breastfeeding or had breastfed recently at the time of the interviews were conducted by using the Functional, Expressive, and Aesthetic (FEA) Consumer Needs Framework to understand user requirements. Based on their insights, the design specifications were established, which guided the design and production of the proposed nursing underwear. In the evaluation phase, a wear trial was implemented, which incorporated a video-based posture analysis for an objective evaluation alongside a survey developed according to the FEA Consumer Needs Framework to collect subjective feedback from the participants. The FEA Consumer Needs Model considers the three criteria of functionality, expressiveness, and aesthetics and is particularly relevant for functional-oriented product development. A review [19] shows that a number of designers have effectively applied this model to develop functional designs and smart garments, thus validating its utility.

### 2.1. User Problem and Needs Identification

A substantial number of studies in the literature have addressed the challenges and needs of breastfeeding mothers. However, there is a notable research gap specifically on Chinese mothers who are breastfeeding. Compared to Western counterparts, Chinese women, in general, have a relatively smaller body size and breasts [20,21], so there may be subtle differences in the demand for breastfeeding posture support products. To address this gap, this study has conducted semi-structured interviews (IRB number HSEARS20240705004). The study recruited twelve Chinese women between 25 and 35 years old who were either currently breastfeeding or had breastfed for at least three months within the past six months at the time of the interviews. The participants all wear a medium-sized top, with a bust measurement between 83 and 90 cm and a waist measurement between 65 and 75 cm. This population is selected as they have real breastfeeding experience and relevant expectations for breastfeeding support products, thus providing authentic and valuable feedback for the nursing underwear design.

Participants with significant health issues or musculoskeletal disorders were excluded from the study. The interviews were conducted via telephone, with each call lasting approximately 15 to 30 min. The interviews were structured according to the FEA Consumer Needs Framework. The functionality aspect explored issues encountered during breastfeeding, requirements for breastfeeding underwear, and perceptions of postural feedback features. The expressiveness aspect examined changes in self-worth and identity during breastfeeding. The aesthetics aspect focused on expectations regarding the style, material, and color scheme of the nursing underwear. The data collected from these interviews were systematically analyzed to identify common issues and needs, which were then used to develop the design guidelines.

### 2.2. Design and Development of Proposed Nursing Underwear

**Figure 2:**
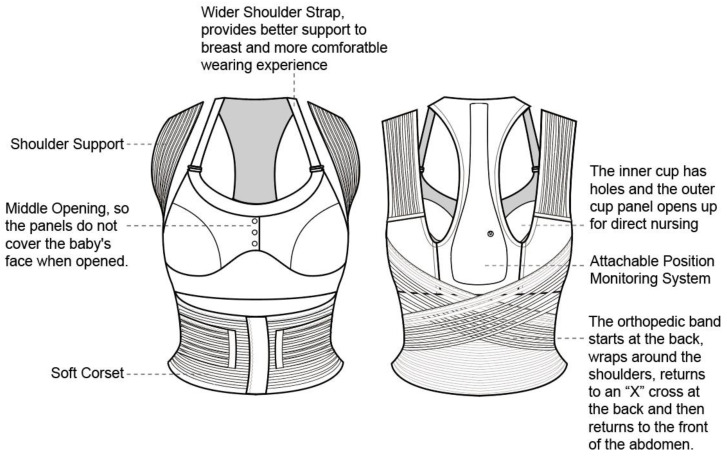
*Construction of prototype design*. Created by the authors.

The design balances breastfeeding accessibility, wear comfort, and functionality after conducting multiple iterations for optimization (Figure 2). The lining material was chosen after several fittings to ensure optimal skin comfort and suitability for extended wear. Shoulder strap widths (1.5 cm, 1.8 cm and 2 cm) and under-bust elastic bands of various configurations (2 cm fold, 3 cm fold, 3 cm single layer, and 4 cm fold) were tested to find the optimal combination of support and aesthetic appeal. Orthopedic elastic band widths of 5 cm, 6 cm, and 7 cm were tested to determine adequate support while ensuring comfort under the arms. Final materials and dimensions were determined through fitting adjustments.

### 2.3. Posture Monitoring System

Among the various sensor technologies available, pressure, optical, flexible, and accelerator sensors, and IMUs are widely utilized for posture monitoring applications. However, due to the need to balance cost, accuracy, portability, and ease of integration into garments, accelerators [22] and flexible bending sensors [23] are selected for this study. 

These formulas clarify the principles and calculation methods of the two sensors.

Voltage divider method to measure the resistance of the flex bending sensor:

(1) $$\mathrm{Voltage}\;\mathrm{Conversion}\text{: }V_{sensor}=\left(\frac{sensorValue}{1023.0}\right)\times 5.0,$$

where $V_{snesor}$ is the voltage across the sensor, the sensor value is the ADC reading (0 to 1023), and 5.0 represents the reference voltage.

(2) $$\mathrm{Resistance}\;\mathrm{Calculation}\text{: }R_{sensor}=(\frac{5.0}{V_{sensor}}-1)\times R_{fixed}$$

where $R_{sensor}$ is the resistance of the bending sensor, $V_{sensor}$ is the measured sensor voltage from the previous equation, $R_{fixed}$ is the known resistance used in the voltage divider.

Tilt angle calculation using accelerometer data:

(3) $$\mathrm{Pitch}\;\mathrm{Angle}\text{: }pitch=arctan(\frac{x}{\sqrt{y^{2}+z^{2}}})\times \frac{180.0}{\pi }$$

(4) $$\mathrm{Roll}\;\mathrm{Angle}\text{: }roll=arctan(\frac{y}{\sqrt{x^{2}+z^{2}}})\times \frac{180.0}{\pi }$$

where *x*, *y* and *z* are the accelerometer readings along the respective axes, arctan is the inverse tangent function (atan in code), and $\frac{180.0}{\pi }$ converts radians to degrees.

**Figure 3:**
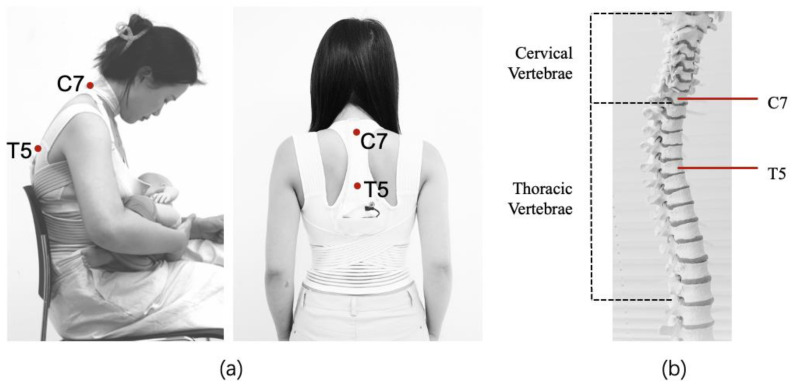
(**a**) *A figure shows the sensor placement on a nursing mother’s body.* Created by the authors; (**b**) *a figure shows the sensor placement on the spinal column chart*. Adapted from Pixabay (n.d.). Retrieved from https://pixabay.com/photos/spine-disc-the-back-disc-prolapse-2539697/ (accessed on 25 October 2024) [24].

After selecting the two types of sensors, their optimal placement was subsequently determined (Figure 3). The cradle hold for breastfeeding was identified as the main reference position for monitoring, as the respondents stated that they usually adopted this position. According to the interview data, when using the cradle or football hold as the position for breastfeeding, the mothers tend to lean against the back of the chair or pillow in the lumbar region. However, placing rigid sensors in the lower back could cause discomfort, particularly since the vertebrae in that area are relatively stable. In contrast, the upper thoracic and cervical spines, and shoulders experience more movement, which require ergonomic attention and awareness. Due to the impracticality of placing sensors on the shoulder areas of breastfeeding garments, the decision was made to place sensors at C7 and T5 to measure the thoracic and cervical flexion angles, respectively.

To implement an effective monitoring system, it is crucial to establish clear criteria for detecting poor posture during breastfeeding, determine the appropriate duration of the posture before triggering an alert, and design a suitable feedback mechanism. There is no consensus in current research on the correct way to implement the cradle hold during breastfeeding; so, based on the conventional breastfeeding position of the mother sitting upright, head bowed, thoracic flexion, and cradling the baby, this article refers to research performed on mobile phone use where the head is leaning forward while seated in order to express the range of joint movement that is used to warn users. Using the craniovertebral angle (CVA) between C7 and the ear [25], head tilts that exceed 30 degrees are identified in [26] as substantially increasing the level of cervical spine stress.

**Figure 4:**
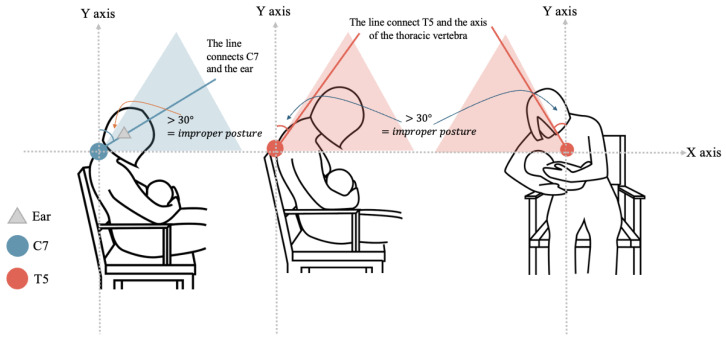
*The standard of improper breastfeeding posture*. Created by the authors.

Due to the complexity of the thoracic spine and the fact that each individual is different, there is a lack of research that clearly indicates how many degrees of thoracic flexion in a seated position will result in significant musculoskeletal pain. However, there is broad consensus on the adverse effects of slumped sitting. In this study, images from five papers [27,28,29,30,31] that define slumped sitting are analyzed, and an approximate angle of 30 degrees is calculated by using a vertical reference line from T5 to T1. Taking T5 as the center of the mass, a vertical reference line was drawn and extended to T1, and the angle was determined to be approximately 30 degrees after averaging the values. Figure 4 provides a visual representation of the criteria for determining “suitable” and “improper” breastfeeding postures, as well as how the specific angle ranges are calculated.

Regarding user interaction, many wearable devices deliver notifications through mobile applications. However, considering the need for mothers to maintain focus on breastfeeding, frequent phone use is impractical. At the same time, the respondents mentioned that their infant might fall asleep during breastfeeding, so it is inappropriate to use an alert mechanism that produces sounds. Consequently, this study opts to incorporate a vibrator into the back of the underwear to provide simple and clear feedback without disturbing the infant. To minimize false alarms, the system trigger was set to activate after 30 s of sustaining an improper posture.

### 2.4. Integration of the Monitoring System

The monitoring system has to span both the thoracic and cervical regions, so coverage is required over a certain area of the body. Thus, the fabric with the embedded sensors must be able to accommodate spinal extension during cervical flexion and thoracic kyphosis. Also, the fabric has to maintain a certain degree of structural rigidity to prevent collapse or wrinkling, which could otherwise compromise the accuracy of the sensors. Materials such as nylon, neoprene, and power net were evaluated for their suitability. Given that the underwear would be worn next to the skin and require frequent laundering, a detachable monitoring system was considered as a potential solution. Initial attempts involved incorporating a patch pocket on the back of the undergarment to allow users to manually insert and remove the sensors. However, this approach was deemed aesthetically unappealing and operationally inconvenient. Consequently, alternative fastening mechanisms, such as snap buttons, magnetic closures, and zippers, were explored to provide a more practical and visually acceptable means of sensor removal and reinsertion. In the end, snap buttons of 0.5 cm in diameter were used.

### 2.5. Evaluation Method

Firstly, a wear trial was subsequently conducted to evaluate the designed prototype. A crossover design was adopted, with participants wearing both a commercially available sample and the designed prototype as two separate conditions. The commercial sample is one of the top-selling nursing bras purchased from Amazon. Each participant underwent the two testing conditions, with the sequence randomized. The participants donned the prototype and simulated breastfeeding by holding a baby doll that weighed 4 kg for 30 min while being video-recorded. They were instructed to use the cradle hold and keep their eye on the mouth of the doll as much as possible. Afterward, the participants were given a twenty-minute break and completed a questionnaire based on the FEA Consumer Needs Framework. Following the break, the participants repeated the breastfeeding simulation but donned the commercial sample under the same posture requirements and duration and were video-recorded again. At the end of the second condition, they completed the same questionnaire.

The sessions were video-recorded to compare and analyze the postural changes under the two different conditions. Two mobile phones were mounted on fixed stands which were placed at the front and side angles of the participant to capture the footage. The video data were analyzed by using Kinovea 0.9.6 software to extract the key metrics, including the neck, shoulder, and thoracic spine positions, to compare the posture changes between the two conditions. Previous studies have demonstrated that using the Kinovea software to extract posture angles from videos or photos is accurate and reliable, with minimal errors that can be considered negligible [32,33,34,35]. In addition, the subjective perceptions of the participants were collected through the questionnaire. The questionnaire covered functionality (posture feedback, corrective functions, wear comfort, and support), expressiveness (self-worth and identity as a mother and woman), and aesthetics (style, design, and color). The feasibility of the product was evaluated by comparing the questionnaire scores under both conditions.

In addition, the posture angle data from the video is compared with the data collected by the sensor in the Arduino system to evaluate the accuracy and stability of the posture monitoring system.

Furthermore, to evaluate the stability and longevity of the device, the donning and doffing test was conducted following international standards, with ASTM D4964 [36] used to assess the tension and elongation properties of elastic fabrics and ISO 12947-2 [37] to evaluate fabric abrasion resistance. The test examined the deformation rate, loose threads, fabric wear level, structural integrity, functionality, and data accuracy of the posture monitoring system (PMS) over 100 cycles.

### 2.6. Data Analysis

For the wear trials, Kinovea software was used to assess head forward flexion, rounded shoulders, and upper thoracic spine angles from the video recordings. The angle of the head forward flexion was measured by using C7 as the center of mass, which was connected to the ear by drawing a horizontal reference line. The rounded shoulder angle was measured by using C7 as the center, which was connected to the acromion by creating a horizontal reference line. The upper thoracic spine angle was measured by using T5 as the center and extended towards T1 and T12. Each breastfeeding simulation was 30 min in length, and data were extracted from 29 frames at equal intervals. To eliminate outliers, frames where the participants were clearly moving, resting, or assuming positions unrelated to breastfeeding were excluded. The mean and standard deviation for each dataset were calculated. Additionally, a Shapiro–Wilk test (*p* > 0.050) was carried out, which indicated that two-thirds of the data were not normally distributed, so non-parametric statistical tests were applied, specifically the Wilcoxon signed-rank test. Statistical significance for all analyses was set at the 95% confidence level. Moments of noticeable relaxation, such as head-raising, neck rotation, or shoulder movement, were also recorded along with their duration. The questionnaire data were divided into five categories based on the question asked: post-breastfeeding fatigue level, wear comfort, effectiveness of posture support, expressiveness, and appearance. The mean for each dataset was then calculated. Significant differences were observed in the mean scores across the five categories when comparing the two conditions. A sample *t*-test was then performed to further verify whether these differences were statistically significant.

Tests to verify the reliability and accuracy of the sensors were as follows. Firstly, the researcher repeated the bending test to obtain angle data corresponding to the change in resistance of the bending sensor. The accelerometer data allowed direct calculation of the tilt angle. Following the experimental protocol, wear tests were fully recorded, and posture angles were measured from video using Kinovea software. The root mean square error (RMSE) was then calculated to quantify the sensor error.

For the donning and doffing test, multiple methods were employed to systematically assess the durability, structural stability, functionality, and data accuracy of the posture monitoring system (PMS). Indicators such as loose threads, fabric wear level, button integrity, fabric structure, and friction discomfort were documented through periodic photography at different cycles (e.g., 1st, 10th, 50th, and 100th) to observe changes in appearance and structural longevity. The deformation rate (%) was calculated by measuring key dimensions (underbust, elastic band length, waistband length, cup length, width, and height). Additionally, PMS functionality was evaluated based on the operation of power, monitoring, and feedback, while data accuracy was determined by comparing actual posture data with sensor outputs, with both metrics rated on a 1–5 scale.

## 3. Results and Discussion

### 3.1. Identified User Needs

Table 1, Table 2 and Table 3 summarize the issues and needs identified from the interviews, with Table 2 focusing on the functional requirements and corresponding design solutions, Table 2 focusing on the emotional and expressive requirements and corresponding design solutions, and Table 3 focusing on the aesthetic requirements and corresponding design solutions.

In terms of functionality, the participants expressed a significant desire to maintain good posture. In addition to preventing musculoskeletal pain, they noted that breast engorgement during lactation often causes unconscious slouching. Furthermore, many caregiving postures involve bending and hunching over. Even when using breastfeeding pillows, they reported that there is still a certain space for movement, which makes it easy to unknowingly maintain poor posture. This supports the findings of Sri Widiastuti et al. [11]. who emphasized that the bra should be comfortable, easy to don and doff, provide adequate breast support, and be machine washable. Additionally, the cup should open easily yet remain secure, thus ensuring that it does not interfere with the infant during breastfeeding. They also suggested that the cup coverage should be larger to avoid exposure during breastfeeding. The results are also in agreement with the findings in [38,39,40], thus suggesting that nursing bra requirements may not differ systematically among women in various countries worldwide. This study has identified the needs of Chinese mothers regarding breastfeeding wearables: the participants prefer that the garment is user-friendly, requires minimal learning or adaptation, aligns with the regular habits of garment wear, and any alerting mechanism should not disturb the baby. The findings support the results in [11] on demands for smart clothing. In terms of expressiveness, the participants expressed frustration with obstacles during breastfeeding, such as pain and body changes. They would like to show that they are beautiful and confident. As for aesthetics, the participants preferred simple designs with attention to detail, thus reflecting their sense of style and elegance.

### 3.2. Final Prototype

**Figure 5:**
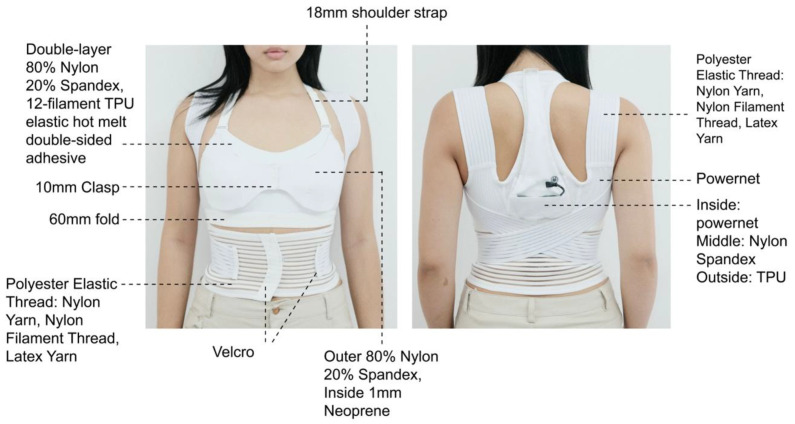
*The Final Protype with Materials Annotation*. Created by the authors.

Based on the interview data, the design prototype was created, which consisted of four main components: a nursing bra, a shoulder posture correction belt, an abdominal binder, and a detachable monitoring system (see Figure 5). To reduce embarrassment during breastfeeding, the cup coverage is slightly larger than typical commercial samples, while the bra features a double-layer design: the inner layer is hollowed out at the nipple area, and the outer layer uses snaps for opening and closing at the center. This design allows the breasts to remain supported and covered during breastfeeding, while the center opening prevents the cup panel from obstructing the face of the infant. The inner layer uses a 12-filament TPU elastic hot melt adhesive to adhere the two layers of high-stretch nylon fabric, thus providing seamless, comfortable elasticity with adequate support. The outer layer of the cup consists of the same fabric with 1 mm neoprene foam for the cups. The 1.8 cm wide shoulder straps and 3 cm double-layer under-bust elastic band provide significant breast support. The shoulder straps are attached to the center of the back, while a 6 cm wide posture correction band extends from both sides, wrapping around the shoulders, crossing at the lower back, and securing at the abdomen with a Velcro fastener. Made with a fishing line elastic band, a breathable and supportive material with spaced fish-line fibers [41], this design effectively promotes shoulder retraction, thus preventing slouching and hunching.

**Figure 6:**
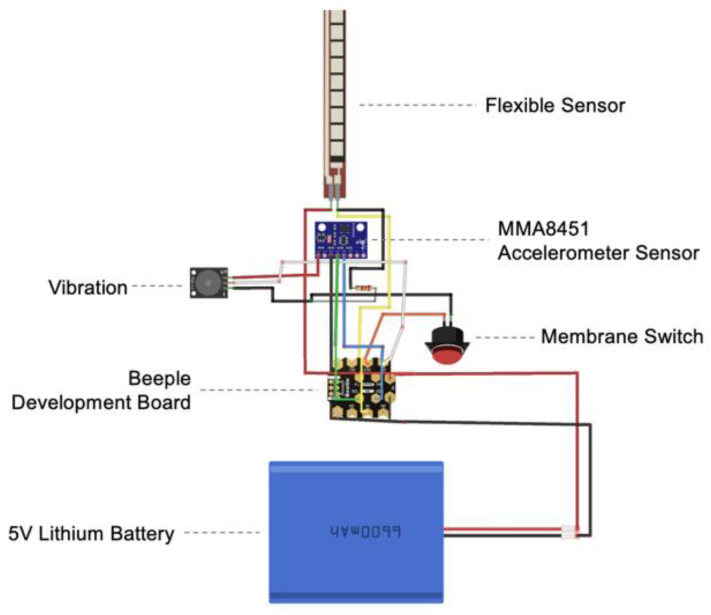
*Circuit board.* Created by the authors.

The abdominal binder is made of the same material, thus tightening the abdomen while providing lumbar support. The monitoring system consists of two parts: the base fabric that houses the electronic components and an outer protective cover. The electronic components include a three-axis accelerometer sensor (MMA8451), flex sensor, Beeple development board, rechargeable 5 V lithium battery, a vibrator, and a push-button membrane switch (see Figure 6). The accelerometer module is placed at the T5 vertebra. The module was chosen over the IMU because it also meets the design target of the study but at a lower cost. The flex sensor is positioned at the C7 vertebra because it is thin and light in weight, thus ensuring that it does not prevent neck movement or affect wear comfort. Beeple, a compact development board designed for wearable technology, is roughly the size of a coin and adapted for conductive stitching. All of the electronic components are connected by using 316 L stainless steel conductive thread embroidered into a 200 GSM power net. The outer protective cover is 80% nylon and 20% spandex, thus providing elasticity, softness, and thickness for a smooth surface of the electronic components. To ensure water and dirt resistance, the outermost layer is TPU fabric. For stretchability, a laser cutting machine was used to create 2 cm wide horizontal and 1 cm wide vertical slits on the TPU cover. The monitoring system can be attached and detached. The prototype’s performance has been evaluated and summarize in Table 4.

The trigger threshold for the flex sensor is set to detect a forward head tilt of 30 degrees, with corresponding Arduino values of around 29,000 Ω. The following is the Arduino code:

currentFlexActive = averageResistance > 29,000

The trigger range of the three-axis accelerometer sensor is between 20 and 60 degrees for forward or lateral tilting. The following Arduino code demonstrates the range within which the accelerometer sensor triggers feedback:

if (filteredY >= −0.85 && filteredY <= −0.35)

if ((filteredX >= 0.25 && filteredX <= 0.7) || (filteredX <= −0.25 && filteredX >= −0.7

To prevent false triggers, the system activates the vibrator only after maintaining a posture within the target range for 30 s. There are three vibration modes based on monitoring outcomes: a 3 s vibration is activated when excessive forward inclination of the neck is detected; a 5 s vibration occurs when the thoracic spine exhibits excessive forward inclination or lateral tilt; and an 8 s vibration is triggered when both the neck and thoracic spine simultaneously exceed their respective acceptable ranges.

### 3.3. Evaluation Result of the Prototype

**Figure 7:**
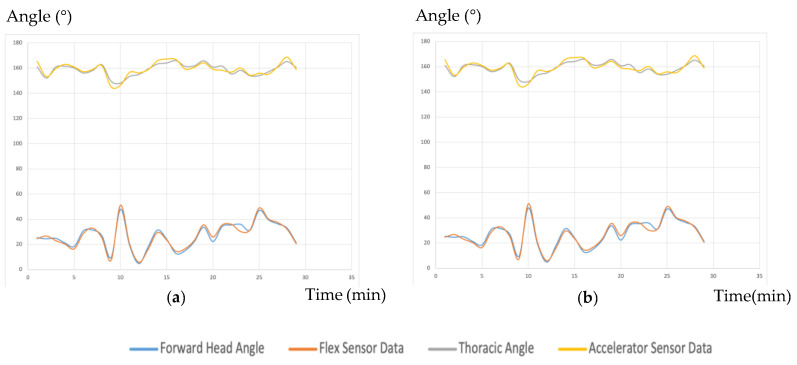
Comparison of the posture angle data from the video and the data from Sensors. (**a**) Subject 001’s data, (**b**) Subject 002’s data.

Figure 7 indicates that the sensor data are stable and accurate, with RMSE values consistently below 5%.

**Figure 8:**
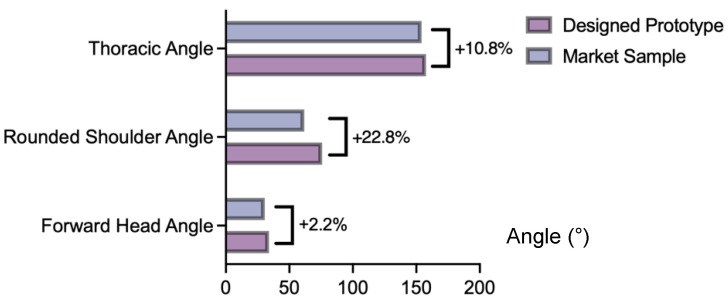
Average posture angle values: commercial vs. prototype.

The impact of the prototype on improving breastfeeding posture, frequency of postural changes, and degree of fatigue after breastfeeding was subsequently evaluated. The average posture angle measurements indicated that when wearing the prototype, the head-forward tilt, shoulder, and thoracic spine angles are larger than those observed with the commercial sample (Figure 8). In particular, Participant 002 showed significantly larger head-forward tilt and shoulder angles, with increases of 25.02% and 52.95%, respectively. These results suggest that the prototype may effectively reduce poor posture during breastfeeding. Additionally, there is a substantial increase in the frequency of relaxation movements, with Participant 001 showing a 60% increase and Participant 002 showing a 175% increase. These demonstrate that the alert function of the prototype encourages users to move and adjust their posture, thereby reducing the risk of musculoskeletal strain from prolonged static postures.

**Figure 9:**
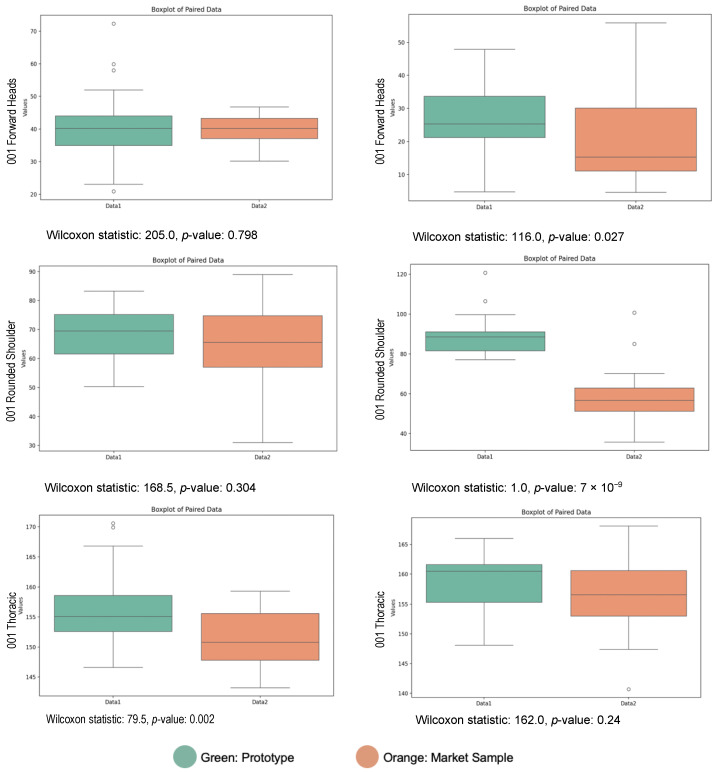
Comparison of postural angle: market sample vs. prototype using Wilcoxon test.

Initial data analysis showed that the postural angle data did not follow a normal distribution, so a Wilcoxon signed-rank test, a non-parametric test for paired data, was used to analyze differences between the commercial sample and prototype. In Figure 9, each boxplot illustrates the distribution of postural angles for each sample. The box shows the interquartile range, the line represents the median, and the whiskers indicate data spread, with outliers marked as points outside the whiskers. Differences in box positions and spreads reflect variations between samples. Results indicate that the thoracic spine angle for Participant 001 and the head-forward tilt and shoulder angle for Participant 002 show statistically significant differences (*p* < 0.05), suggesting the prototype may positively influence these angles. While other angles did not reach significance, they exhibit a trend toward higher values with the prototype, indicating a potential alignment with a healthier posture. This variation in significance may be influenced by the small sample size and individual differences among participants.

**Figure 10:**
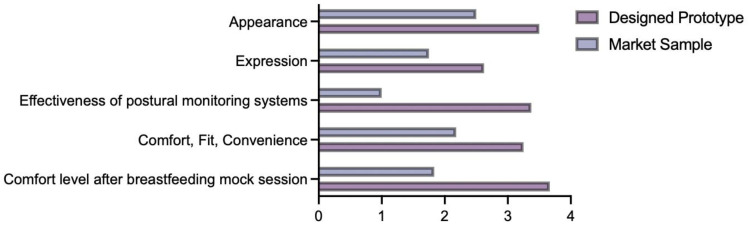
Averaged data based on questionnaires.

In addition to the objective measurements, subjective feedback from the questionnaire provided further data. As shown in Figure 10, the participants reported a 49.17% reduction in post-breastfeeding fatigue when wearing the prototype compared to the commercial sample. In terms of wear comfort, fit, and convenience, the prototype scores 49% higher than the commercial sample, while expressiveness and aesthetics are 50% and 40% higher, respectively. The practicality and effectiveness of the monitoring system received an approval rating of 3.375/4. The paired sample T-test results showed that these differences are statistically significant (*p* < 0.05), thus further validating the observed differences in the mean values. Both participants reported significant reductions in fatigue levels, even though not all posture angle data showed substantial changes. This could be attributed to the increased frequency of relaxation movements and posture shifts during the trials, which are in agreement with the findings in the study by Akkarakittichoke et al. [42].

The donning and doffing test results (Table 5) show that the fabric of the garment remains intact through the first 50 cycles, with slight wear and structural loosening appearing by the 100th cycle. The fabric’s deformation rate gradually increases, reaching 5.1% after 100 cycles and causing slight friction discomfort. The posture monitoring system (PMS) maintains functionality and high data accuracy through 50 cycles, with a slight drop in data accuracy to level 4 by the 100th cycle. Overall, the underwear prototype demonstrates good durability over repeated use.

### 3.4. Research Significance

The innovativeness of the study prototype is found in its ability to monitor and correct posture, and breastfeeding accessibility within a single garment, an approach not thoroughly explored in previous research. The findings suggest improved breastfeeding experiences, maternal self-efficacy, and infant health. Additionally, the product may support commitment to breastfeeding, which influences fertility rates and sustainable population growth. Beyond breastfeeding, this design could benefit women with posture issues from sedentary lifestyles, thus offering a reference for future development of smart, functional underwear.

### 3.5. Limitations

Despite the encouraging results, several limitations in this study must be acknowledged. First, the sample size limits the generalizability of the findings. Broad conclusions cannot be drawn from such a small cohort of participants. Furthermore, the short simulation period may not fully capture posture changes during extended breastfeeding sessions. More complex posture changes and interference are likely in real-life conditions, where users engage in a wider range of activities while wearing the underwear. Consequently, the wear comfort, effectiveness, and stability of the prototype for practical applications require further investigation.

### 3.6. Future Work

Future research could focus on collecting more comprehensive breastfeeding posture data to inform the development of a posture classification model. This model could be implemented as a mobile application to track and record posture data, incorporating a customizable feedback mechanism that allows users to adjust vibration intensity, duration, and frequency. Additionally, developing a Finite Element (FE) model to assess the structural performance of the undergarment would provide further insights into its postural support effectiveness, enabling optimization of design parameters. For a more robust evaluation, future studies could also integrate motion capture technology to accurately capture three-dimensional posture changes during breastfeeding. Furthermore, using a dynamic dummy that simulates realistic infant movements would closely approximate real breastfeeding conditions, thereby enhancing the validity of wear trials.

## Figures and Tables

**Table 1:** Functional requirements and corresponding design solutions based on interview data.

Problem	Need	Solution
Shoulder Pain Neck Pain Back Pain Breast and Nipple Pain Significant changes in breast size before and after breastfeeding Chest hunching due to breast pain and enlargement Hunching in various positions while caring for the baby Flaccid abdomen post-partum Lack of portability of breastfeeding pillows Need for positional adjustments even when using a breastfeeding pillow Inadequate support provided by breastfeeding underwear Potential obstruction of the baby’s face by the outer layer of breastfeeding underwear, thus affecting breastfeeding	Posture support Comfortable Safe Non-irritating to the skin Easy to don and doff Construction reduces exposure during breastfeeding Supports the breasts without being overly constricting Easy-to-use cup opening and closing mechanism The cup panels should not block the view of the nipple during breastfeeding Posture monitoring must not affect the infant Easy-to-use monitoring function Machine washable	Orthopedic shoulder support elastic band Abdominal support elastic band Seamless cup lining Double-layered cups for breastfeeding accessibility Central closure on the outer layer Neoprene cup padding and nylon outer layer, soft and elastic Wide elastic under bust band and wide supportive shoulder straps Integrated posture monitoring system on the back for real-time feedback Removable monitoring system, the remaining garment is machine washable Water–dirt-proof thermoplastic polyurethane (TPU) protection on the exterior of the monitoring system, which does not require cleaning

**Table 2:** Emotional and expressive requirements and corresponding design solutions based on interview data.

Problem	Need	Solution
Impact on self-confidence from body changes during breastfeeding Emotional stress caused by body pain Embarrassment due to breast exposure during breastfeeding	Improve self-confidence through better posture and body image Emotional comfort and ease of use during breastfeeding Reduced embarrassment during breastfeeding by minimizing breast exposure	Posture support elastic band to improve body image Double-layered cup design with extensive internal coverage to reduce breast exposure during breastfeeding

**Table 3:** Aesthetic requirements and corresponding design solutions based on interview data.

Problem	Need	Solution
Unattractive or overly utilitarian design that diminishes self-esteem	A simple, modern style that complements functionality and enhances appearance	Minimalistic design with clean lines and thoughtful detailing

**Table 4:** The prototype’s performance parameters.

Parameter	Specification
Functions	Breastfeeding posture monitoring and real-time feedback
Feedback Mechanism	The vibration alerts operate in three modes: 3 s for neck monitoring, 5 s for thoracic spine monitoring, and 8 s for combined issues.
Reliability	Supports up to 500 monitoring cycles without performance degradation
Accuracy	Lower than 5%
Response Time	50 ms
Signal Noise Level	Lower than 5%
User Comfort	Lightweight design (<200 g) with breathable, stretchable, and hypoallergenic fabric
Battery	Up to 8 h of continuous use,
Operating Conditions	Temperature: −10 °C to 50 °C; Humidity: ≤85%
Washability	Detachable sensor components, machine washable fabric

**Table 5:** The result of the donning and doffing test.

Test Cycles	Loose Threads	Fabric Wear Level (1–5)	Structural Integrity (Button)	Fabric Structure (1–5)	Deformation Rate (%)	Friction Discomfort	Functionality Integrity of the PMS (1–5)	Data Accuracy of the PMS (1–5)
1	None	5	Intact	5	0	None	5	5
10	None	5	Intact	5	1.2	None	5	5
50	Few	5	Intact	4	3.5	None	5	5
100	Few	4	Few Detached	4	5.1	Slight	5	4

## Data Availability

The data presented in this study are available upon request from the corresponding author (J.Y).
